# Forecasting Progressive Trends in Keratoconus by Means of a Time Delay Neural Network

**DOI:** 10.3390/jcm10153238

**Published:** 2021-07-22

**Authors:** Marta Jiménez-García, Ikram Issarti, Elke O. Kreps, Sorcha Ní Dhubhghaill, Carina Koppen, David Varssano, Jos J. Rozema, on behalf of The REDCAKE Study Group

**Affiliations:** 1 Department of Ophthalmology, Antwerp University Hospital (UZA), Drie Eikenstraat 655, 2650 Edegem, Belgium; isarti.ikram@gmail.com (I.I.); Sorcha.NiDhubhghaill@uza.be (S.N.D.); carina.koppen@uza.be (C.K.); 2 Department of Medicine and Health Sciences, University of Antwerp, 2000 Antwerp, Belgium; 3 Department of Ophthalmology, Ghent University Hospital, 9000 Ghent, Belgium; elke.kreps@ugent.be; 4 Department of Ophthalmology, Tel Aviv Sourasky Medical Centre, Tel Aviv 64239, Israel; davidva@tlvmc.gov.il

**Keywords:** keratoconus, corneal ectasia, keratoconus progression, Scheimpflug tomography, supervised machine learning, neural network, artificial intelligence, corneal imaging

## Abstract

Early and accurate detection of keratoconus progression is particularly important for the prudent, cost-effective use of corneal cross-linking and judicious timing of clinical follow-up visits. The aim of this study was to verify whether a progression could be predicted based on two prior tomography measurements and to verify the accuracy of the system when labelling the eye as stable or suspect progressive. Data from 743 patients measured by Pentacam (Oculus, Wetzlar, Germany) were available, and they were filtered and preprocessed to data quality needs. The time delay neural network received six features as input, measured in two consecutive examinations, predicted the future values, and determined the classification (stable or suspect progressive) based on the significance of the change from the baseline. The system showed a sensitivity of 70.8% and a specificity of 80.6%. On average, the positive and negative predictive values were 71.4% and 80.2%. Including data of less quality (as defined by the software) did not significantly worsen the results. This predictive system constitutes another step towards a personalized management of keratoconus. While the results obtained were modest and perhaps insufficient to decide on a surgical procedure, such as cross-linking, they may be useful to customize the timing for the patient’s next follow-up.

## 1. Introduction

Keratoconus (KC) is a progressive, ectatic corneal disorder presenting with central or paracentral stromal thinning associated, among others, with corneal protrusion, structural changes, and transparency loss [1,2,3]. The condition has a multifactorial etiology, attributable to a genetic predisposition, eye rubbing, or biomechanical factors [4], and a cascade of biomechanical decompensation triggered by a focal change in corneal elasticity is thought to be responsible for KC progression [5]. Given this complex context, it is hardly surprising that clinicians cannot form a consensus on a common definition of clinical progression. Nevertheless, definitions for progression have been established based on the repeatability of the clinical devices, which determines the minimum change measurable at a pre-established significance level, keeping in mind that repeatability is worse in keratoconic corneas [6]. This approach was recently introduced in the Pentacam ABCD progression display [7].

To complicate matters further, progression in KC presents considerable interindividual variability: from cases with significant progression in 3 months [8] to cases that remain stable for more than a decade [9]. These clinical variations, as well as the effectiveness of corneal cross-linking (CXL) [10], have led to a renewed interest in assessing the risk factors for a faster KC evolution [11]. Although stabilization via CXL may be performed based on other factors (e.g., an unstable refraction that requires regular, costly updates of the refractive correction), many national CXL protocols require proof of KC progression to qualify for reimbursement [12]. Progression is typically established by inviting the patient for a follow-up visit about 6 months later. If progression occurs, the patient receives CXL; if not, another follow-up visit is planned. The obvious disadvantage with this approach is that, typically, the patient loses visual quality before being treated. Thus, early detection of KC progression is as useful clinically as establishing the diagnosis of KC itself. At present, age and maximum keratometry are mainly used to schedule the next follow-up [11], but additional features to stratify progression risk would be of added value.

The concept of a “suspect KC” has gained relevance in recent years [13], and a similar concept could be put into practice for KC progression [14]. Identifying such “suspect progressive KC” would be valuable to determine whether certain patients might benefit from closer follow-up or a fast-track CXL [8,15], but this would require a forecast of KC evolution. Such predictive tasks are well suited for machine learning, which has been widely used in KC since the 1990s [16] to distinguish normal from KC corneas [17,18,19,20]. Forecasting systems based on neural networks have also been extensively used in other fields with long-term time series of balanced data, such as speech or stock markets [21], and occasionally in medicine [22], though this approach has not yet been used in KC.

This work presents a novel neural-network-based system to classify KC as “stable” or “suspect progressive”. It uses longitudinal tomographical data series, which are generally short, irregularly spaced, or with a variable number of follow-ups and, as previous approaches, considers that surpassing the noise threshold is the absolute minimum to define a progressive trend [7].

## 2. Materials and Methods

### 2.1. Patients

The Retrospective Digital Computer Analysis of Keratoconus Evolution (REDCAKE) is a retrospective multicenter observational study organized through the European Vision Institute Clinical Research Network (EVICR.net). A large longitudinal dataset of keratoconic eyes with at least 2 corneal tomographies (separated at least 5 months) was created; 906 KC patients were enrolled, 743 (1155 eyes) measured with Pentacam (Oculus Optikgeräte GmbH, Wetzlar, Germany) and 163 with Galilei (Ziemer Ophthalmic Systems AG, Port, Switzerland). Since Galilei data did not include information on measurement quality, only patients measured with Pentacam were considered in this study.

The REDCAKE dataset includes patients across the spectrum of the disease, ranging from forme fruste KC (contralateral eye without topographical or slit-lamp signs of an eye diagnosed as KC [13,23]) to severe cases. The patients were recruited in tertiary centers in Europe and Israel, and each underwent a comprehensive ophthalmological examination, including tomography, by an experienced cornea specialist. These included the earlier forms of KC identified using the Belin–Ambrósio Display Deviation (BADD), the presence of risk factors such as genetic predisposition or eye rubbing, and clinical expertise. Manifest KC was diagnosed according to well established criteria (corneal steepening, stromal thinning, inferior–superior asymmetry, Vogt striae, etc.) [9]. Ocular surgeries or comorbidities, contact lens change, fluorescein instillation, and systemic disease except allergies were considered grounds for exclusion. The baseline findings for these patients are described elsewhere [24].

REDCAKE was designed and carried out in compliance with the tenets of the Declaration of Helsinki, and voluntary informed consent was obtained from all patients where required by local legislation. Ethical approval was granted by the Institutional Review Board of all the centers (ClinicalTrials.gov ID NCT03235856).

### 2.2. Corneal Parameters Included

A previous study evaluated the monotonicity, repeatability, and consistency of more than 250 Pentacam variables to establish which would be the most suitable parameters to describe KC progression according to those criteria [25]. That analysis included the latest available tomographic parameters of Pentacam, such as BADD, the ABCD classification variables, and pachymetry profiles.

Based on the top 10 parameters from that list (see Table S1), 6 nonredundant, potentially platform-independent parameters were selected: age, known to affect KC progression [11,26]; the average keratometry in a 3 mm area around the maximum keratometry (KmaxZonalMean3mm) [27]; the steepest radius (RsF) and best fit sphere over an area of 8 mm (BFSF) of the front surface; and the average radius of the back surface (RmB) and LOGIK [18], which is based on the elevation maps of both corneal surfaces and the minimum pachymetry. None of the parameters included were based on a single corneal point.

### 2.3. Suspect Progressive KC Definition

The minimally detectable change is directly linked to the repeatability of the parameters provided by Pentacam in KC, which was recently reported [6]. Repeatability is defined as r = S_w_ × 1.96 × √2, where S_w_ is the within-subject standard deviation, and the difference between two measurements for the same subject is expected to be <2.77 × S_w_ for 95% of the pairs of observations [28]. Double-sided 95% confidence intervals (95 CI) were created as [−r, +r]. This approach is similar to the one proposed in an ABCD progression display [7], although this uses a two-sided 95 CI.

The same criterion for defining a suspect progressive KC was used for both the measured and the predicted values of the 2nd follow-up. Only changes in the direction of clinical progression were considered (e.g., a significant increase in KmaxZonalMean3mm or LOGIK or a significant decrease in any of the radii or BFSFs). If any of the 5 variables (excluding age) reached a level outside the 95 CI of their corresponding baseline value in the clinical direction of progression, the triplet was labelled as suspect progressive. In any other case, the triplet was labelled as stable.

### 2.4. Data Preprocessing

Data preprocessing was required to ensure the adequate training of the system. The techniques are specified here to ensure reproducibility:

#### 2.4.1. Data Maximization Design: Triplets

**Figure 1:**
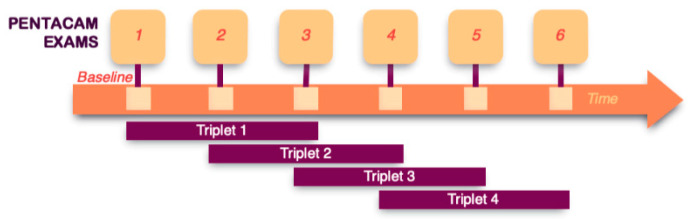
Triplets of consecutive measurements to maximize data availability. Data maximization was performed, creating triplets of consecutive measurements for those eyes with more than 3 examinations. The triplets were classified as no errors whether all the measurements were marked as OK by the Pentacam software. For triplets with error, only 1 measurement was allowed to be of less quality (marked yellow by Pentacam).

**Figure 2:**
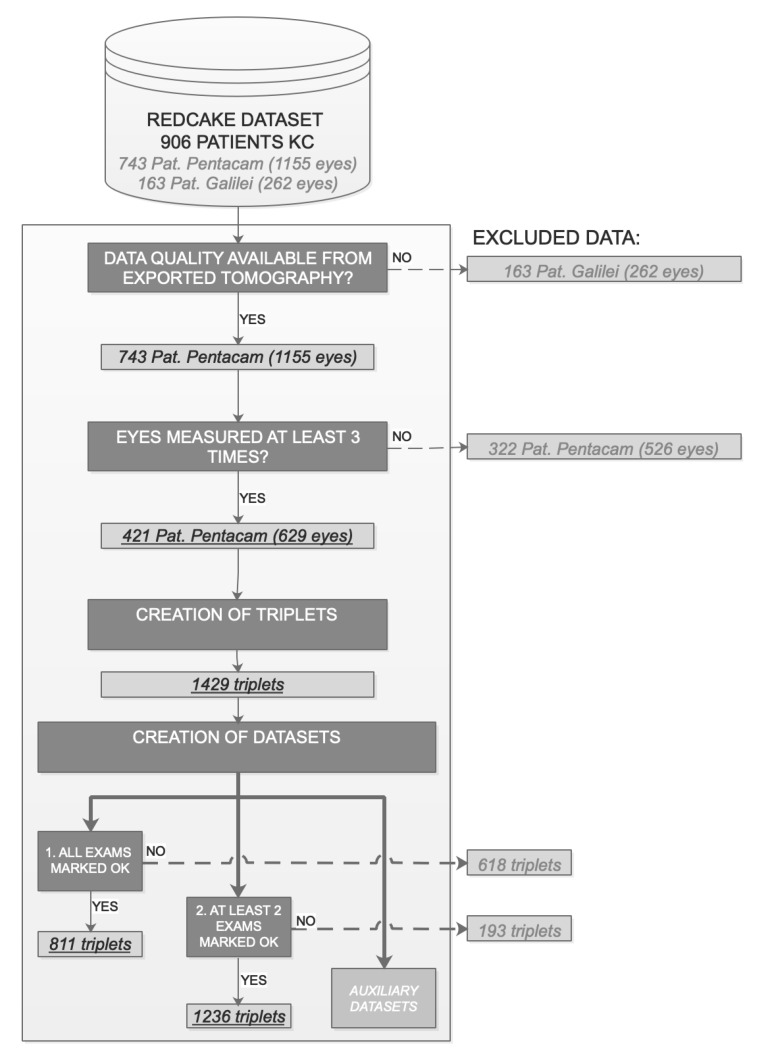
Data filtering. Creation of datasets. Final number of patients, eyes and triplets are highlighted in underlined italics.

Since the method aims to forecast KC progression based on 2 prior measurements, it is possible to maximize data availability by dividing the follow-up series of each eye into triplets of 3 consecutive examinations (Figure 1). This increases the number of progression patterns captured and, thus, the stability of the prediction system. A total of 1429 triplets were created for a total of 629 eyes with 3 or more examinations (Figure 2).

The underlying theoretical concept is similar to data augmentation [29], where in this case, the additional data (triplets) were generated by selecting 3 consecutive examinations when additional follow-ups were available. These triplets may be considered a series of real patients that make only 3 visits to the same ophthalmologist. In our analysis, these data units will be referred to as “triplets”, rather than “eyes” or “patients”.

#### 2.4.2. Data Quality and Robustness to Errors

Data quality is of great importance in machine learning applications [30]. To this end, only triplets with the 3 exams marked “OK” by the Pentacam software were considered observations. Severe cases often fail to get an “OK” by the software, so this choice led to the most severe cases being excluded, while the remaining ones could act like outliers that could influence the training. Hence, any triplet classified as severe KC at the baseline (LOGIK > 3.5, average maximum keratometry of 72.6 ± 12.4D) was also excluded. After the quality check, *n* = 811 triplets remained.

To verify how the quality of the data may affect the performance of the system, the predictive system was also trained and tested with a second dataset (*n* = 1236 triplets) in which 1 of the 3 exams included in the triplet was allowed to be of lower quality (marked in yellow by the Pentacam software).

Finally, to ascertain whether having a more time-balanced dataset would lead to a more stable configuration, auxiliary smaller datasets were tried, formed by triplets whose periods between examinations were restricted (e.g., between 6 and 9 months).

Under these restrictions, our data did not present missing values.

#### 2.4.3. Noise Reduction and Normalization

Both noise reduction and normalization were used to facilitate training; different techniques have been used previously for this purpose [31]. Since the purpose of the system is a binary classification based on the 95 CI, the following nonlinear noise reduction algorithm was applied: the baseline value is not modified, since it is considered the reference; for the values of the 1st and 2nd follow-ups, 3 different strategies are applied depending on the measured value. (1) For values inconsistent with the clinical direction of progression, the difference with the baseline value is reduced by a factor of 5. (2) For values inside the 95 CI with a trend consistent with clinical progression, the factor selected is 2 since that trend might contain information about future progression. (3) Values consistent with the clinical direction of progression and outside the 95 CI are not scaled. This process does not affect the decision of the algorithm since the 95 CI is the boundary (anything inside it is considered stable), and values consistent with progression outside the 95 CI are not transformed. For example, imagine a triplet of real values for KmaxZonalMean3mm (44; 43.5; 45)D. For that variable, 95 CI = 0.72D and the clinical progression trend is increasing. The system calculates the differences to the baseline value (44D): Δ = (0; −0.5; 1)D. The 2nd delta (−0.5D) is opposite to the clinical progression direction, so it is reduced by a factor of 5; the 3rd delta is higher than the 95 CI, so it is not scaled Δ_transformed_ = (0; −0.1; 1)D. The triplet is transformed to (44; 43.9; 45)D. This triplet shows less variability than the original but, with the same classification, is suspect progressive. Only 1 variable was shown for simplicity, but an equivalent procedure is applied for each of the variables (excluding age).

Finally, data were normalized using the Euclidean norm and denormalized after training to present the results.

### 2.5. Network Architecture

The multi-input, multi-output time delay neural network (TDNN) uses the baseline variables (6, so multi-input) and the 1st follow-up (6) visit to predict the 2nd follow-up (6), the desired output.

The network was configured with an input layer, a hidden layer, and an output layer. Due to the complexity of the problem, the hidden layer was configured with 25 neurons. The optimal number of neurons was empirically selected after multiple trials. The Levenberg–Marquardt optimization algorithm was used to train the network with a sigmoid activation function. An open-loop TDNN provides a finite dynamic response to time series, in this case a 1-step-ahead prediction based on 2 previous examinations. These networks take the sequence of the exams into consideration (order matters), but the exact timing between examinations is not considered. To create a timeframe for the prediction, “age” was used as an input of the system, and the predicted value for that variable was used as the location in time for the prediction. Subsequently, linear interpolation was used to calculate the predicted values at the real date of the 2nd follow-up, thereby allowing comparisons between the measured and the predicted status.

### 2.6. Data Split: Training and Test Datasets

**Figure 3:**
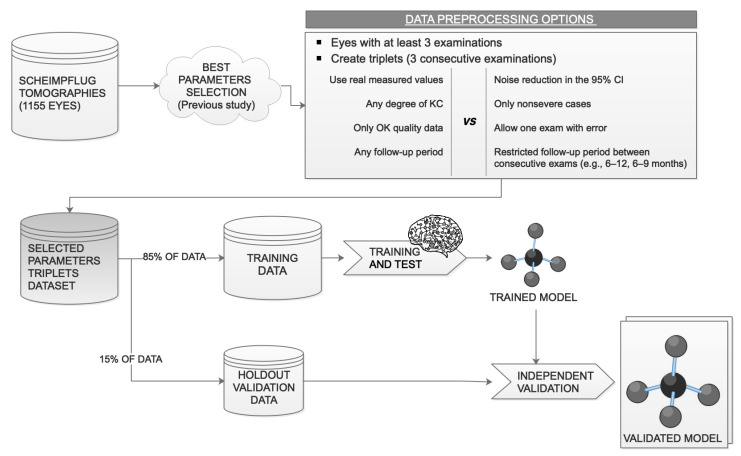
Data usage. After the data preprocessing, 85% of the data are used for training and 15% are kept for an independent holdout validation of the trained model. KC, keratoconus; 95 CI, 95% confidence interval.

The available data were divided into training and validation sets as follows: 85% of the data (Figure 3) were randomly selected for network training (internally subdivided into 70% for training, 15% for testing, and 15% for internal validation during training), and 15% of the triplets were used as an independent validation set for the trained model (holdout validation).

Triplets belonging to the same original eye have different baseline values, different confidence intervals, and different desired outputs, and all of them are nonlinearly transformed to reduce noise. Based on these facts, data split was performed at the level of triplets and not at the level of patients or eyes. Performing it at another level would also create different group sizes in each iteration since not all the eyes have the same number of follow-ups.

### 2.7. Data Analysis

To verify the stability, the TDNN was retrained 10 times with random training and test observations. The sensitivity, specificity, positive predictive value (PPV), and negative predictive value (NPV) of the system were calculated using the independent holdout validation dataset for each of the iterations.

Matlab R2020b (MathWorks Inc., Natick, MA, USA) was used to configure the neural network, and JMP Pro 15 (SAS Institute Inc., Cary, NC, USA) was used for statistical analysis; alpha 0.05 was considered the cut-off value for significance.

## 3. Results

The REDCAKE dataset includes longitudinal Scheimpflug series of 906 patients (26% female, 74% male). After the filtering (Figure 2), 629 eyes from 421 patients measured with Pentacam remained with a mean age of 26.8 (6.4) years. Average maximum keratometry, minimum pachymetry, and BADD grouped by ABCD classification of these 629 eyes can be found as Table S2.

Using the definition above, 423 of the 811 (52%) triplets were classified as stable. The other 388 triplets were classified as suspect progressive, of which 156 triplets presented changes exceeding the 95 CI in one parameter, 79 triplets in two parameters, 48 triplets in three parameters, 62 triplets in four parameters, and 43 triplets in all five parameters.

To verify the behavior and reproducibility of the TDNN under various data quality restrictions, the system was trained and tested with two different datasets. The first was exclusively composed of good-quality triplets (*n* = 811), while in the second, one measurement in the triplet was allowed to be of lower quality (*n* = 1236). Table 1 shows the sensitivity, specificity, PPV, and NPV of 10 consecutive stable training/validation iterations. The upper rows show the results of a TDNN trained and tested with high-quality triplets; the lower rows show the results for the second dataset. On average, the TDNN proved a sensitivity of 70.8% and a specificity of 80.6% when trained and tested with high-quality triplets. No significant difference was found in sensitivity, specificity, PPV, and NPV when training with triplets with or without error (Wilcoxon test, *p* > 0.05).

When the triplets used were restricted to those whose periods between examinations were restrained (to create a more time-balanced dataset), the training process was less stable.

**Figure 4:**
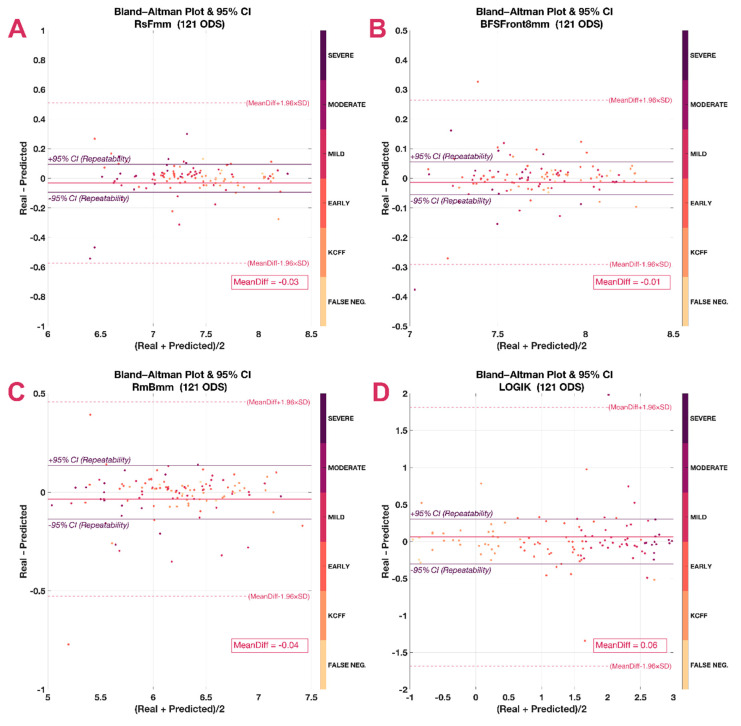
Bland–Altman plots. Besides the mean difference (MeanDiff) and its confidence intervals (dashed line), they include the repeatability (continuous line and italics). (**A**) Steepest radius of the front surface in mm (RsF), (**B**) average radius of the back surface in mm (RmB), (**C**) best fit sphere of the front surface considering an 8 mm area (BFSF), (**D**) logistic index for keratoconus, LOGIK.

Using Bland–Altman plots, it was seen that most predicted values fell within the 95 CI of the real value (repeatability), but there were also some outliers (Figure 4). These outliers did not necessarily correspond with more severe cases at the baseline, but were present in all stages (indicated by marker colors).

**Figure 5:**
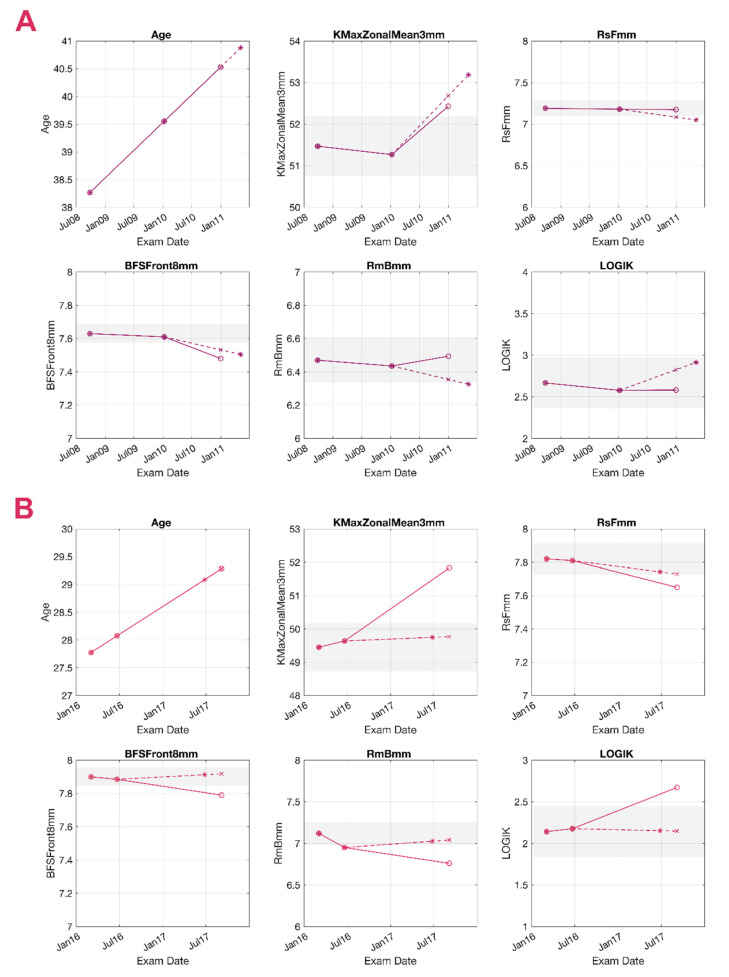
Prediction of a true-positive (TP) and a false-negative (FN) case. Grey areas represent the 95% confidence interval (95 CI) around the baseline value. Circles (o) represent the measured values in 3 consecutive examinations, and the asterisk (∗) represents the output of the system. The cross (×) is calculated by linear interpolation and aligned to the 2nd follow-up date. Continuous lines represent the measured trend, and the dashed lines represent the predicted trend. Measurements within the 95 CI are presented with the noise reduction applied. (**A**) TP, 38 years old. Significant change was found in the average keratometry in a 3 mm area around the maximum curvature point (KmaxZonalMean3mm) and the best fit sphere (BFS) in both the prediction and the real values. (**B**) FN, 27 years old. Although all 5 variables presented significant change, the system classified the eye as stable (none of the predicted variables reached the 95 CI). BFSF: best fit sphere of the front surface considering an 8 mm area, RmB: average radius of the back surface in mm, RsF: steepest radius of the front surface in mm.

**Figure 6:**
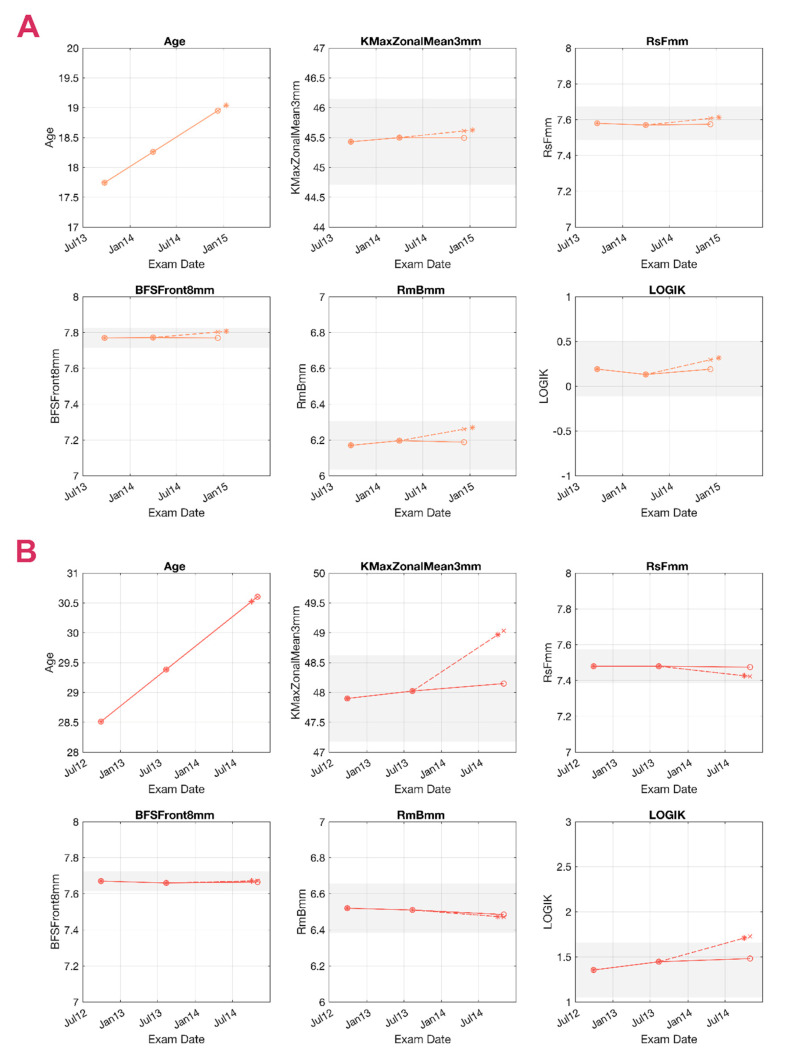
Prediction of a true-negative (TN) and a false-positive (FP) case. Grey areas represent the 95% confidence interval (95 CI) around the baseline value. Circles (o) represent the measured values in 3 consecutive exams, and the asterisk (∗) represents the predicted output. The cross (×) is calculated by linear interpolation and aligned to the 2nd follow-up. Continuous lines represent the measured trend, and the dashed lines represent the predicted trend. Measurements within the 95 CI are presented with the noise reduction applied. (**A**) TN, 17 years old. The system predicted stability, so there was no significant change, which coincided with the real measurements. (**B**) FP, 28 years old. The system predicted a significant change in the average keratometry in a 3 mm area around the maximum curvature point (KmaxZonalMean3mm) and LOGIK, but the measured values remained stable within the 95 CI. BFSF: best fit sphere of the front surface considering an 8 mm area, RmB: average radius of the back surface in mm, RsF: steepest radius of the front surface in mm.

Figure 5 and Figure 6 present examples of a true-positive (TP), a false-negative (FN), a false-positive (FP), and a true-negative (TN) case as detected by the TDNN, representing KC cases of various stages at the baseline and different ages. Since follow-up times vary considerably between patients, the TDNN forecasts the likely date of the patient’s return (first panels in Figure 5 and Figure 6), for which it will forecast the five corneal parameters. In reality, however, the patient may show up earlier or later than this forecasted follow-up date, so linear interpolation or extrapolation was used to allow comparing the forecasted values with the real parameter values at the second follow-up. In the graphs, solid lines represent the real measured trends, while dashed lines correspond with the forecasts. The dashed line contains two representative points: the “∗” represents the estimated date, while the “×” is the linearly interpolated value for the actual follow-up date. For example, the longitudinal data of patient A in Figure 5 is used to predict the second follow-up. The system predicts the value for the six variables; May 2011 is considered the timeframe for the prediction, and all the “∗” are located on that date. To allow comparisons, the variables were calculated by linear interpolation on January 2011 (the date when the second follow-up took place) and are represented as “×”.

## 4. Discussion

From clinical and economic perspectives, two critical decisions must be made in KC: when to schedule the next visit and whether a patient would benefit from CXL, which can halt KC progression in 90%–95% of the treated cases [32,33] in a cost-effective way [34,35]. CXL, however, is not without risks [36], and national reimbursement protocols often require proof of progression prior to treatment. The current methods for identifying the risk for KC progression [37] are insufficient to truly stratify risk and personalize the follow-up visits, which are mainly scheduled based on patient age. A reliable forecast of the evolution of the ectasia would therefore be a valuable tool to plan follow-ups and interventions.

This study presented a predictive system that classifies KC as either stable or suspect progressive based on two prior tomographies, which is, to the best of our knowledge, the first system based on a TDNN developed for this purpose. On average, the sensitivity and specificity of the system were modest and probably insufficient to decide on a fast-track CXL. However, with an average PPV of 71.4% and NPV of 80.2%, the system may be useful to personalize the follow-up visits, advancing the next visit for suspect progressive cases and delaying the timing for those predicted as stable.

As expected, the percentage of triplets categorized as suspect progressive here (48%) is higher than the rates of clinically meaningful progression in recent studies [38,39]. We also note that had one-sided confidence intervals been used [7], 1.96 would have to be replaced by 1.64 in the formula, narrowing the 95 CI and increasing the percentage of suspect progressive cases. Since not overtreating patients and not creating unnecessarily advanced appointments are crucial in the public health system, we opted for the most conservative approach. A previous study also suggested that larger confidence intervals—using 3SD—were more appropriate in KC progression to increase specificity [40].

Innumerable combinations of variables could have been considered. Our selection comes from a previously published list of candidate parameters [25], reduced to keep the minimum number of potentially platform-independent variables that included information from both corneal surfaces. Additionally, this selection complies with the specifications of the global consensus to define KC progression [41]. Well-known indices, such as A and B (from the ABCD progression display) or BADD, while sensitive and specific for KC progression [12,42], were not included as they are specific to Pentacam, and their underlying calculations are not public. More than half of the KC showed progression by an ABCD progression display earlier than by the standard criterion based on maximum keratometry in a recent publication [12]. In this work, we wanted to go one step further and verify not only whether progression occurred but also whether progression could happen shortly.

The potential role of family history, concomitant allergies, eye rubbing, or biomechanical parameters is interesting, but their repercussion on the system’s accuracy remains unknown. Unfortunately, biomechanical devices are less widespread than corneal tomographers, and creating a high-quality longitudinal dataset of sufficient volume is unrealistic at this time. The same limitation may be expected in a similar model implemented for post-LASIK ectasia. Exploring how the current model behaves on these particular ectasias may be explored in the near future.

In our study, being more permissive with the measurement quality did not significantly affect the results, suggesting either robustness to errors or a limitation due to data availability. The observation that a more time-balanced dataset did not improve stability may support a data limitation; training with fewer triplets (around 400) led to less stable training, even if they were better balanced in time.

Although using data from both eyes can be considered appropriate in asymmetric diseases, such as keratoconus [43], this choice may require justification. TDNNs are specialized neural networks to work with longitudinal data, which are usually highly correlated, and they have likewise proven their efficiency with highly correlated variables [44]. Hence, as the present study does not involve regressions or correlations, as a nonlinear transformation was applied to the triplets for noise reduction, and as the 95 CIs used here derive from a previous study, it is possible to include both eyes from the same patient, provided that they both meet the quality restrictions.

The limitations of our study also need to be addressed. First, two examinations are required to predict the trend, while predictions based on a single examination would be preferable. Second, robustness to errors needs further investigation since getting high-quality measurements is not always possible in KC, especially in the advanced stages, though these cases are often not candidates for CXL anyway due to more severe corneal thinning. We also observed that the patients older than 35 years old tended to be overly classified as suspect progressive, possibly due to their under-representation within the sample. Finally, with the aim of not introducing further uncertainties, the prediction was linearly interpolated to the date of the second follow-up visit (see Figure 5 and Figure 6), while aligning all the values to a fixed date (e.g., 6 months apart from the first follow-up) would probably be more clinically relevant. This study did not include a healthy control group; however, this should not be seen as a real limitation. Many systems have been specifically developed for KC detection [19,20,23]. The system presented here is further in the clinical path and requires a confirmed KC (or at least a suspicious case), and it is not to be used in the normal population. The inclusion of a healthy group would only complicate the task for which it was designed, unbalancing the dataset in favor of the stable cases.

## 5. Conclusions

Aiming for a more personalized approach to KC management is, in our opinion, the way to go, and previous studies have been carried out with that objective in mind [33,45,46]. Here, we presented another step forward, a predictive system with the prospect of a platform-independent generalization. The availability of sufficiently high-quality data is a crucial limiting factor in accomplishing this objective. The inclusion of biomechanical variables in the model as additional nonredundant parameters would undoubtedly be of clinical interest; therefore, compiling enough longitudinal biomechanical data is a major challenge for us to address in the near future. These facts emphasize the relevance of collaborative research projects, such as REDCAKE, especially in pathologies with a low prevalence, such as KC.

## Figures and Tables

**Table 1:** Sensitivity, specificity, and positive and negative predictive values of 10 iterations considering both options: training/test with and without error permitted.

OPTION 1: Triplets without Error (*n* = 811; 121 Used for External Validation)											
	1	2	3	4	5	6	7	8	9	10	AVG
SENS	66.0%	77.5%	70.4%	74.1%	69.6%	75.6%	66.0%	60.0%	82.6%	66.0%	70.8%
SPEC	84.5%	76.5%	80.6%	73.0%	85.3%	73.7%	83.1%	84.5%	80.0%	84.5%	80.6%
PPV	75.0%	62.0%	74.5%	71.7%	74.4%	63.0%	73.3%	73.2%	71.7%	75.0%	71.4%
NPV	77.9%	87.3%	77.1%	75.4%	82.1%	83.6%	77.6%	75.0%	88.2%	77.9%	80.2%
**OPTION 2: Triplets with 1 Error Allowed (*n* = 1236, 169 Used for External Validation)**											
	**1**	**2**	**3**	**4**	**5**	**6**	**7**	**8**	**9**	**10**	**AVG**
SENS	70.1%	69.6%	70.6%	66.7%	68.1%	75.0%	77.3%	71.6%	77.8%	73.4%	72.0%
SPEC	82.9%	83.3%	82.1%	84.9%	83.5%	75.2%	72.8%	74.7%	67.9%	72.2%	78.0%
PPV	81.3%	78.6%	80.0%	72.4%	75.4%	67.1%	64.6%	68.8%	59.0%	69.9%	71.7%
NPV	72.3%	75.8%	73.4%	81.1%	77.9%	81.7%	83.3%	77.2%	83.7%	75.6%	78.2%

SENS = sensitivity, SPEC = specificity, PPV = positive predictive value, NPV = negative predictive value, AVG = mean.

## Data Availability

Data might be available under reasonable request pending the agreement of all the contributing centers. Original codes might be available under reasonable request to the corresponding authors.

## Supplementary Materials

The following are available online at https://www.mdpi.com/article/10.3390/jcm10153238/s1: Table S1: Top 10 parameters, Table S2: Eye classification.
