# Supplementary material for: Forecasting Progressive Trends in Keratoconus by Means of a Time Delay Neural Network
Source: J Clin Med. 2021 Jul 22;10(15):3238. doi: 10.3390/jcm10153238 (PMC8347247; doi:10.3390/jcm10153238)
Supplement: Supplementary file 1 [file jcm-10-03238-s001.zip › jcm-1283469-conversion SI/Supplementary Materials- Table S1-Top10 parameters.pdf]

Supplementary Material. Table S1. TOP 10 parameters. Coefficient of variation (repeatability), monotonicity and consistency (% of eyes) of the best Pentacam variables, with partial scores, and global score.

				VALUES			PARTIAL AND GLOBAL SCORES				
**RANK**	**VARIABLE**	**SURFACE**	**TYPE**	**REPEATABILITY**	**MONOTONICITY**	**CONSISTENCY**	**REPEATABILITY**	**MONOTONICITY**	**CONSISTENCY**	**GLOBAL SCORE**	
**1**	KMaxZonalMean5mm	F	CUR	0.42%	0.47	73.54%	4	2	18	***24***	*Redundant. 3mm area is chosen since it might be less influenced by peripheral measurements*
**2**	**KMaxZonalMean3mm**	F	CUR	0.48%	0.47	73.79%	6	1	17	***24***	
**3**	KMaxZonalMean4mm	F	CUR	0.45%	0.47	72.82%	5	4	25	***34***	
**4**	K2F (D)	F	CUR	0.61%	0.46	73.06%	11	6	21	***38***	*Redundant. Radius is chosen since it does not require assumptions about keratometric index*
**5**	**RsF (mm)**	F	CUR	0.49%	0.45	72.82%	7	9	23	***39***	
**6**	**BFSFront8mm**	F	BFS	0.27%	0.43	73.06%	1	27	20	***48***	
**7**	**RmB (mm)**	B	CUR	0.86%	0.43	75.73%	17	31	10	***58***	
**8**	**LOGIK**	-	IND	3.58%	0.47	76.21%	47	3	9	***59***	
**9**	RmF (mm)	F	CUR	0.39%	0.45	70.39%	3	13	44	***60***	*Slightly redundant*
**10**	RMin (mm)	F	CUR	0.84%	0.45	72.09%	16	14	33	***63***	*One-point parameter*

SURFACE: F (Front), B(Back).

TYPE: CUR (Curvature), BFS (Best Fit Sphere), IND (Index)

VARIABLES: KmaxZonalMean- average keratometry in areas of 3, 4 or 5mm around the point of maximum keratometry, K2- steepest keratometry (D), Rs- steepest radius (mm), BFS-Best Fit Sphere, Rm- Mean radius (mm), Rmin- Minimum radius (mm)
