# Supplementary material for: Forecasting Progressive Trends in Keratoconus by Means of a Time Delay Neural Network
Source: J Clin Med. 2021 Jul 22;10(15):3238. doi: 10.3390/jcm10153238 (PMC8347247; doi:10.3390/jcm10153238)
Supplement: Supplementary file 1 [file jcm-10-03238-s001.zip › jcm-1283469-conversion SI/Supplementary Materials- Table S2-Eyes Classification.pdf]

Supplementary Material. Table S2. ABCD classification, number of eyes máximum keratometry (Kmax), mínimum pachymetry (Pmin) and Belin-Ambrósio Deviation (BADD). Mean (SD).

ABCD CLASSIFICATION	NUMBER	Kmax	Pmin	BADD
A0B0C0	101	*45.04 (1.93)*	*525.08 (20.08)*	*1.94 (0.86)*
A0B0C1	46	*46.42 (2.03)*	*475.50 (10.81)*	*3.02 (1.04)*
A0B0C2	13	*45.96 (1.26)*	*444.46 (6.02)*	*3.93 (0.98)*
A0B1C0	18	*47.69 (2.07)*	*523.44 (16.16)*	*3.59 (0.97)*
A0B1C1	18	*48.10 (2.00)*	*473.39 (10.02)*	*4.12 (0.64)*
A0B1C2	2	*46.95 (0.45)*	*447.00 (1.00)*	*5.01 (0.36)*
A0B2C0	22	*48.41 (2.10)*	*517.18 (15.13)*	*4.69 (1.14)*
A0B2C1	15	*49.31 (2.73)*	*473.80 (12.60)*	*5.74 (1.16)*
A0B2C2	3	*48.00 (1.39)*	*441.67 (9.10)*	*5.52 (0.12)*
A0B3C1	1	*50.30 (0.00)*	*482.00 (0.00)*	*6.49 (0.00)*
A0B3C2	1	*45.20 (0.00)*	*434.00 (0.00)*	*8.61 (0.00)*
A0B4C0	1	*50.60 (0.00)*	*502.00 (0.00)*	*7.82 (0.00)*
A0B4C3	1	*51.50 (0.00)*	*378.00 (0.00)*	*8.41 (0.00)*
A1B0C0	2	*48.80 (0.60)*	*506.00 (5.00)*	*2.06 (0.37)*
A1B0C1	2	*47.40 (0.10)*	*486.00 (4.00)*	*1.74 (0.49)*
A1B1C0	3	*50.93 (0.82)*	*502.00 (8.29)*	*3.90 (0.28)*
A1B1C1	6	*51.17 (1.08)*	*473.83 (6.04)*	*3.95 (0.58)*
A1B1C2	4	*49.23 (1.12)*	*438.75 (8.41)*	*4.47 (1.00)*
A1B2C0	17	*49.38 (1.47)*	*520.53 (15.36)*	*4.74 (1.17)*
A1B2C1	19	*50.25 (1.49)*	*472.21 (12.71)*	*5.59 (0.83)*
A1B2C2	11	*50.23 (1.75)*	*436.09 (10.25)*	*5.88 (0.93)*
A1B3C1	2	*48.35 (0.95)*	*475.00 (2.00)*	*7.63 (0.25)*
A2B1C1	1	*49.60 (0.00)*	*467.00 (0.00)*	*5.14 (0.00)*
A2B1C2	1	*50.70 (0.00)*	*439.00 (0.00)*	*5.45 (0.00)*
A2B2C0	22	*53.38 (2.68)*	*518.00 (15.08)*	*5.52 (1.16)*
A2B2C1	36	*52.88 (2.48)*	*469.08 (10.95)*	*6.19 (0.92)*
A2B2C2	18	*52.78 (2.19)*	*435.28 (10.55)*	*6.65 (1.21)*
A2B3C0	14	*52.85 (2.36)*	*510.43 (17.53)*	*7.23 (0.89)*
A2B3C1	30	*54.03 (2.30)*	*469.23 (9.84)*	*7.71 (1.15)*
A2B3C2	13	*53.89 (2.24)*	*431.54 (9.76)*	*7.95 (0.96)*
A2B4C0	9	*54.38 (3.26)*	*507.00 (11.22)*	*7.84 (0.96)*
A2B4C1	27	*55.51 (2.85)*	*466.81 (9.22)*	*9.13 (1.07)*
A2B4C2	19	*55.16 (2.53)*	*434.74 (9.85)*	*9.91 (1.21)*
A2B4C3	2	*54.90 (1.60)*	*384.00 (4.00)*	*12.62 (1.63)*
A3B3C1	1	*55.10 (0.00)*	*487.00 (0.00)*	*6.95 (0.00)*
A3B4C0	3	*59.70 (2.45)*	*501.00 (2.94)*	*10.89 (0.54)*
A3B4C1	20	*57.30 (2.53)*	*472.55 (12.32)*	*10.57 (1.60)*
A3B4C2	17	*58.58 (2.66)*	*427.71 (13.65)*	*11.15 (1.27)*
A3B4C3	4	*58.63 (4.34)*	*386.75 (15.93)*	*12.84 (2.13)*
A4B4C1	16	*63.06 (4.57)*	*473.00 (10.00)*	*12.25 (1.86)*
A4B4C2	39	*62.82 (5.41)*	*433.13 (13.63)*	*12.91 (2.77)*
A4B4C3	28	*64.54 (6.29)*	*384.57 (14.20)*	*15.40 (4.09)*
A4B4C4	1	*81.00 (0.00)*	*230.00 (0.00)*	*41.05 (0.00)*
**Total**	**629**			
